# Food Acquisition in the Geography of Brazilian Obesity

**DOI:** 10.3389/fpubh.2020.00037

**Published:** 2020-03-06

**Authors:** Camila Elisa Alves, Glenio Piran Dal' Magro, Keitiline R. Viacava, Homero Dewes

**Affiliations:** ^1^Agribusiness Interdisciplinary Research Center, Federal University of Rio Grande do Sul, Porto Alegre, Brazil; ^2^Decision Making Laboratory (DM.Lab), São Paulo, Brazil; ^3^Department of Biophysics, Biosciences Institute, Federal University of Rio Grande do Sul, Porto Alegre, Brazil

**Keywords:** supply chain, agribusiness, short supply chain, food insecurity, food security, malnutrition, hunger, agri-food

## Abstract

Overweight and obesity are a worldwide pandemic with geographic differences. Possible explanations include variable access to food and its quality, dietary habits of the populations, behavioral patterns, and characteristics of the food markets. This study aimed to examine the acquisition of food in the different regions of Brazil and to relate it with the geography of Brazilian obesity. We used data provided by a Brazilian official organ, which gathers periodic data on the household food acquisition. Descriptive statistics and multidimensional scaling techniques were used to ascertain the similarity of food acquisition among populations in the Brazilian states. High levels of overweight and obesity occur in all states (>44%), especially in the southern half of the country (>54%). We found differences in the food acquisition patterns throughout the country. Furthermore, we identify that states with similar dietary patterns have similar population levels of overweight and obesity, demonstrating a possible relationship between the food supply models and these food insecurity manifestations as expressed in the individual health. However, the occurrence of regional singularities suggests that the food supply model constitutes only one of the multiple variables that compete for diversity in the Brazilian regional distribution of obesity and overweight. We found that socioeconomic conditions influence nutritional misalignment in the geography of Brazil. Our results show that overweight and obesity have a higher occurrence in middle age (35–79 years), and it is more present in females. Moreover, women with lower education and lower incomes have higher levels of overweight and obesity, an association of unhealthy food intake with poverty. In men, obesity is more frequent in those with more schooling and higher incomes. Based on the widely variable geographical characteristics of the distant states of Brazil, we conclude that overweight and obesity go beyond an individual lifestyle and access to quality food, and is more related to a complex framing of factors, like schooling, age, sex, income, feeding patterns, food markets, and anthropological circumstances.

## Introduction

Human health is connected to food security—a concept that involves availability, accessibility, and sufficient quantity for the proper use of food. The most widely used definition refers to the access of all people, at any time, to sufficient and adequate food. Food must provide energy and nutrients to maintain an active and healthy life (1, 2).

We live in a state of global alertness about food security (3). Even with the increase in global availability and the decrease in food prices, there are still other influences outside the individuals, which can determine their dietary patterns and, consequently, cause a nutritional misalignment. In addition, food security can also be jeopardized by environmental factors, which influence the behavior of individuals. As environmental factors, we refer to all those external to the individual—social, economic, political, biophysical, and demographic.

Local factors with characteristics that reinforce behaviors related to obesity make up an obesogenic environment. In this sense, understanding the environmental factors that contribute to obesity is an essential step in characterizing and supporting eventual interventions in preventing collective overweight and obesity (4).

Overweight and obesity come from abnormal or excessive fat accumulation, according to the World Health Organization (5). Therefore, a healthy diet can help prevent them. Obesity is considered a risk factor in developing chronic diseases, such as cardiovascular problems, hypertension, and diabetes, with mortality more related to obesity than malnutrition. Chronic diseases are the main causes of mortality in the world, accounting for 63% of deaths in 2008, the year of the last data of this present work (6). The theme of obesogenicity has been on the agenda of priorities of researchers, social rulers, food industry, and society, in general.

It is not new that the indices of overweight and obesity have assumed high proportions around the world, and it is considered a major threat to public health (6, 7). This pandemic, as it has been considered, is increasing in almost all countries (8). About 2.5 billion people, over 18 years, were identified with overweight or obesity in 2016, representing approximately 40% of the world population (6). This reality may come to harm the development of populations, as much as poverty.

Among the explanations for the problem are, although not restricted, the habits and dietary patterns of the populations. Different factors can affect access to food, such as economic, socio-demographic, cultural, and environmental aspects. All these factors are, in principle, measurable and sensitive to observation, including information about behaviors of peoples located in the same geographic space. Understanding the environmental characteristics of the sites that present high rates of the nutritional disorder is a fundamental tool for public health.

The objective of this study is to examine the determinants of the geography of Brazilian obesity and overweight, taken into account that Brazil presents a marked regional heterogeneity in terms of biophysical, socioeconomic, and historic and cultural diversity.

In a previous study, we found that food acquisition in the Brazilian states is geographically diversified. The heterogeneity observed emphasizes Brazilian agribusiness development and underscores the influence of the food supply chains on the regional food patterns (9). In this work, we want to check whether this food acquisition diversity and the regional socioeconomic conditions are related to the spatial distribution of the excess weight and obesity in the Brazilian population.

## Materials and Methods

This study was based on the analysis of data obtained in the Family Budget Survey (POF). We were limited to data corresponding to the years 2008–2009, the most updated set published by the Brazilian Institute of Geography and Statistics (IBGE) so far (10).

The POF was organized in partnership with the Ministry of Health and seeks to present a profile of the living conditions of the Brazilian population. Therefore, through the measurement of consumption, expenditures, and income of individuals, the POF has the purpose of assessing relevant themes for the creation of public policies. We decided to use the POF microdata for a more objective view on the prevalence of obesity and overweight in Brazil. From this, we initiated an analysis to identify patterns that could explain the spatial distribution of overweight and obesity in Brazilian populations in a comprehensive way.

The microdata of the POF consist of codified numerical data, classified accordingly to the nature of the information and whether it is individual or familiar. We used the acquired food amounts and the anthropometric and socioeconomic data. These numbers were downloaded on TXT archive. From there, we opened the dataset with EXCEL software. Next, we read the sequential numerical data using a dictionary of variables provided in the same URL page of IBGE, which allowed us to build a table with the desirable variables, which were analyzed by Statistical Package for Social Sciences (SPSS, version 18). The data presented in this work are those obtained after this step.

The present investigation uses the anthropometrical perspective to access food insecurity. In this sense, obesity and overweight were quantified, using the specific indicator of body mass index (BMI). To calculate the BMI (weight in kg/square of height in m), the microdata of the POF was used. From this base, the values of body weight and height for the calculation were extracted, whereby we created a new variable. The nutritional status variable was categorized as underweight (BMI < 18.5 kg/m^2^), eutrophic (BMI between 18.5 and 24.9 kg/m^2^), overweight (BMI between 25 and 29.9 kg/m^2^), and obesity (BMI ≥ 30 kg/m^2^).

Weight and height measurements were carried out by the institution's agent during home visits. The residents who were not present did not obtain records of anthropometric measurements. After calculating the BMI and the classification of the individual's nutritional status, we verified the percentage of adults aged over 25 with obesity or overweight in each of the 27 Brazilian federative units (states). It is noteworthy that BMI is considered a less effective index for the measurement of obesity in children, justifying our choice to analyze only individuals aged 25 years or older (11). In adults, BMI is considered the most useful measure, and can be used to estimate the prevalence of overweight and obesity within a population and the risks associated with it (5).

To analyze the Brazilian food acquisition, we worked with data on quantities acquired of food and beverages, which refer to all acquisitions for household consumption, both monetary and non-monetary. To standardize, all items were converted to the metric unit kilogram (kg). Concerning food types, the data were classified into the 17 groups pre-established by IBGE (2010): (1) cereals and legumes, (2) flour and pasta, (3) coconuts, chestnuts, and nuts, (4) vegetables, (5) fruits, (6) sugars and products of confectionery, (7) salts and condiments, (8) meats and offal, (9) fish, (10) canned and preserves, (11) poultry and eggs, (12) dairy products, (13) bakery, (14) industrialized meats, (15) beverages and infusions, (16) oils and fats, and (17) prepared foods.

Moreover, we decided to implement multidimensional scaling to investigate the similarity of food acquisition among Brazilian states. Multidimensional scaling consists of an interdependence technique that allows mapping objects, considering the distance or similarity between the elements analyzed (12). The data regarding the food acquisition consist of the medians of quantity in kilograms for each food group acquired in the year 2008–2009 per Brazilian federative unit. The medians were chosen because they eliminate the influence of outliers, avoiding possible distortions in the result that the extreme values can cause. For the elaboration of the proximity matrix, we considered the significant Pearson correlations at the 1% level (prioritizing the highest values among the federative units). The data were processed by the software Statistical Package for Social Sciences (SPSS, version 18), and the alpha level (α) of 5% was considered as the significance criterion.

We grouped the Brazilian federative units according to their similarity in the median food quantity acquired from each of the 17 pre-established IBGE food groups. This process allowed us to make judgments of consumers regarding their preferences in multidimensional space.

In addition, it is known that there is an impact regarding social class on nutritional patterns (13–16). Thus, we used socioeconomic data to characterize the relationship between obesity and dietary patterns. We investigated whether there are differences in the spatial distribution of overweight due to gender or age of the individuals interviewed. For the participants' ages we used the time in years from the date of birth of the respondent to the date of the interview. Moreover, schooling and income data were also related with BMI. In addition, to determine schooling, we used the last grade level at which the respondent was enrolled. For income, values in the Brazilian currency referring to the per capita remuneration of the interviewees were used.

## Results

**Figure 1:**
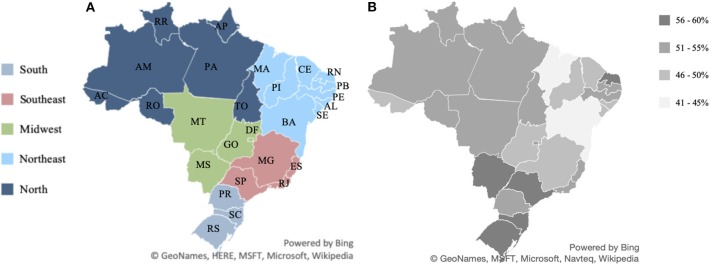
Geographic macro-regions of Brazil and overweight and obesity indices in each of the Brazilian states. Source: Data derived from the Brazilian Family Budget Survey—Brazilian Institute of Geography and Statistics (10). **(A)** Map of the five Brazilian macro-regions. **(B)** Map of the spatial division of overweight indices in Brazilian federative units.

The geopolitical subdivision of Brazil in macro-regions and the spatial variation of excess weight in the population are shown in Figure 1. The map of the five Brazilian macro-regions is shaded based on geographical delimitations. The map of the spatial division of overweight indices in Brazilian federative units is shaded according to the percentage of the population with overweight and obesity. The two images show the similarity between the spatial distributions of the prevalence of obesity in the five macro-regions of the country.

**Figure 2:**
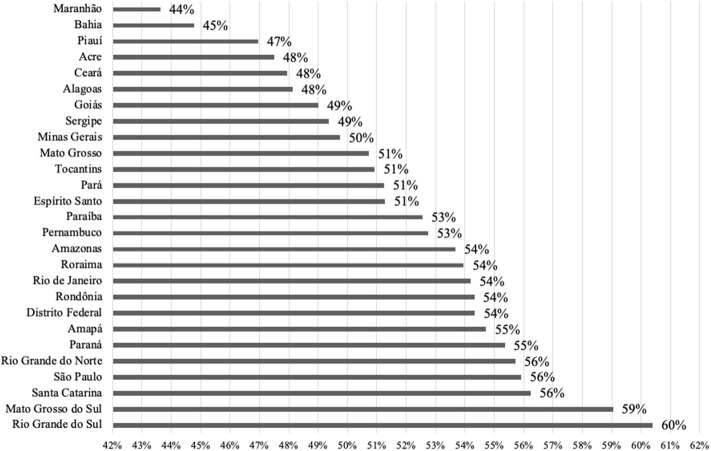
Percentage of overweight plus obese people in each of the Brazilian states. Source: Data derived from the Brazilian Family Budget Survey—Brazilian Institute of Geography and Statistics (10).

**Figure 3:**
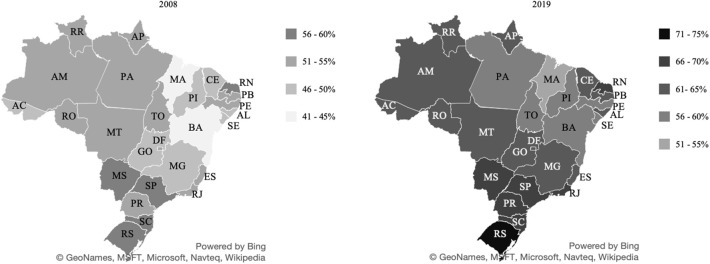
Percentage of people with overweight and obesity in the Brazilian states in 2008/09 and 2019. Source: Data derived from the Brazilian Family Budget Survey—Brazilian Institute of Geography and Statistics (10) and Food and Nutritional Surveillance System (17).

In our study, we considered 27 federative units and five macro-regions of Brazil (Table 1). In Figure 2 one can see the percentage of overweight and obese people in each Brazilian state. Figure 3 shows that the spatial distribution of overweight and obesity in the Brazilian federative units in 2008 was similar to that of the year 2019. In this way, we understand that our analysis, which is limited to the available data from 2008, is still pertinent to configurating a picture of the country at later times.

**Table 1:** Macro-regions and the respective federative units of Brazil.

**Macro-regions**	**Federative units**
South	Paraná (PR), Santa Catarina (SC), Rio Grande do Sul (RS)
Southeast	Minas Gerais (MG), Espírito Santo (ES), Rio de Janeiro (RJ), São Paulo (SP)
Midwest	Mato Grosso (MT), Goiás (GO), Distrito Federal (DF), Mato Grosso do Sul (MS)
Northeast	Maranhão (MA), Piauí (PI), Ceará (CE), Rio Grande do Norte (RN), Paraíba (PB), Pernambuco (PE), Alagoas (AL), Sergipe (SE), Bahia (BA)
North	Acre (AC), Amazonas (AM), Rondônia (RO), Roraima (RR), Amapá (AP), Pará (PA), Tocantins (TO)

*Data derived from the Brazilian Institute of Geography and Statistics (10)*.

**Figure 4:**
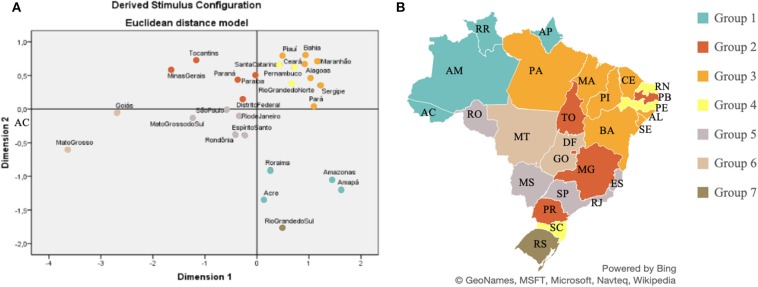
Similarities of household food acquisition in the Brazilian states, based on the multidimensional scale, in the period 2008–2009. Source: Data derived from the Brazilian Family Budget Survey—Brazilian Institute of Geography and Statistics (10). **(A)** Euclidean distance arrangement between Brazilian federative units according to food acquisition. **(B)** Colors indicate the states grouped according to their respective, similar patterns of food acquisition.

Figure 4 shows how spatial distribution occurred in terms of home food acquisition in Brazil. Specifically, the image reflects the similarities, regarding annual per capita Brazilian household food acquisition. The map is shaded according to the proximity between the states, referring to the respective Pearson correlations. The states that belong to each respective group are shown in Table 2.

**Table 2:** Brazilian federative units grouped according to respective food acquisition in 2008–2009.

**Group**	**Federative units**
Group 1	Acre (AC), Amapá (AP), Amazonas (AM), Roraima (RR)
Group 2	Tocantins (TO), Paraná (PR), Distrito Federal (DF), Paraíba (PB) e Minas Gerais (MG)
Group 3	Ceará (CE), Maranhão (MA), Bahia (BA), Piauí (PI), Pará (PA), Alagoas (AL) e Sergipe (SE)
Group 4	Rio Grande do Norte (RN), Pernambuco (PE) e Santa Catarina (SC)
Group 5	Espírito Santo (ES), São Paulo (SP) Rio de Janeiro (RJ) Mato Grosso do Sul (MS) e Rondônia (RO)
Group 6	Mato Grosso (MT) e Goiás (GO)
Group 7	Rio Grande do Sul (RS)

*Data derived from the Brazilian Family Budget Survey—Brazilian Institute of Geography and Statistics (10)*.

**Figure 5:**
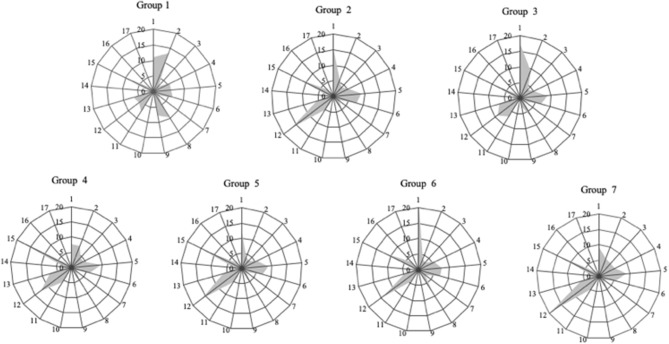
Food acquisition profiles of the seven groups of Brazilian states, categorized according to the 17 IBGE food groups, in the period 2008–2009. Source: Data derived from the Brazilian Family Budget Survey—Brazilian Institute of Geography and Statistics (9). IBGE Food Groups: 1—cereals and pulses; 2—starch flour and pasta; 3—coconuts, nuts, and walnuts; 4—vegetables; 5—fruits; 6—sugars and confectionery; 7—salts and condiments; 8—meat and offal; 9—fish; 10—canned and preserves; 11—birds and eggs; 12—dairy products; 13—bakery products; 14—processed meat; 15—beverages and infusions; 16—oils and fats; 17—prepared foods.

The Euclidean distances between Brazilian federative units were calculated according to the food amount purchased. Data on median food acquisition refer to the amount of food per capita per household purchased in each Brazilian federative unit. Figure 5 shows the composition of the diets in the seven state groups formed. Each group is formed by the states that have similar diet patterns.

Dietary patterns are not the only explanation for the spatial division of overweight/obesity. Thus, it makes sense to analyze other factors that may be associated with overweight, such as socioeconomic factors (age, gender, income, education). We identified that obese individuals have a median age higher than the other individuals analyzed. Contrastingly, eutrophic individuals have a lower median age. In Brazil, the largest number of overweight and obese individuals was found in the age group of 50–79 years. Moreover, the nutritional situation of Brazilian women deserves great attention because they are more prominent according to our data, both in the underweight and obesity levels.

**Figure 6:**
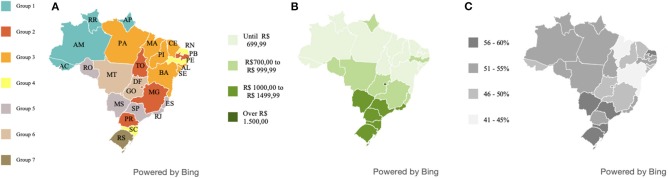
Geographic distribution of the states with similar dietary patterns and their respective average *per capita* income per household and level of obesity and overweight in Brazil, period 2008–2009. Source: Data derived from the Brazilian Family Budget Survey—Brazilian Institute of Geography and Statistics (10). **(A)** Map of the spatial division of groups of Brazilian federative units with a similar dietary pattern. **(B)** Map of the average income of individuals by Brazilian federative unit. **(C)** Map of spatial division of overweight indices in Brazilian federative units.

We marked the Brazilian states according to the respective average *per capita* income. Further, we confront the individual states with the food groups and with the overweight and obesity data. From this analysis, we can see that states with higher average income per individual tend to have higher rates of overweight (Figure 6).

**Figure 7:**
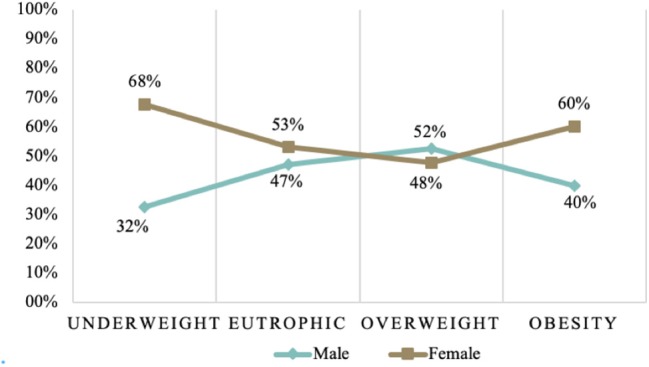
Nutritional status of adults over 25 years of age, separated by sex, in the period 2008–2009 in the Brazilian population. Source: Data derived from the Brazilian Family Budget Survey—Brazilian Institute of Geography and Statistics (10).

**Figure 8:**
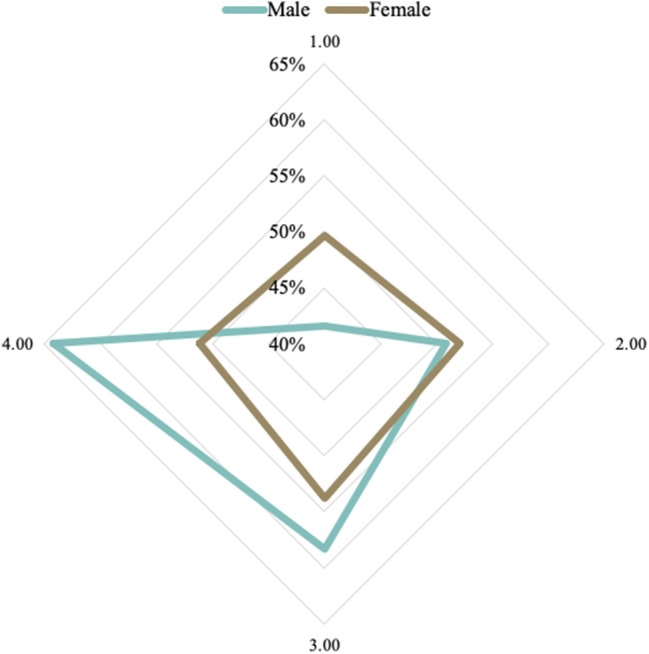
Prevalence of overweight and obesity by sex, according to the income levels in the Brazilian population, in the period 2008–2009 (level 1, the lowest; level 4, the highest). Source: Data derived from the Brazilian Family Budget Survey—Brazilian Institute of Geography and Statistics (10).

Sex is among the socioeconomic variables that positively affect the rates of overweight and obesity Figure 7 shows that women present more obesity indexes than men do. Figure 8 shows the sex of overweight/obese individuals in each of the income quartiles. Quartiles are the division of the country's total families into four groups of equal size and grouped according to the *per capita* household income.

**Figure 9:**
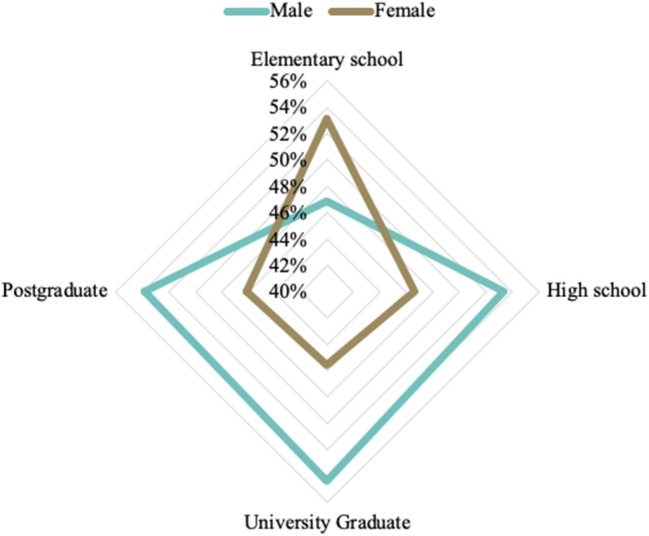
Prevalence of overweight and obesity according to schooling levels and sex in the Brazilian population, in the period 2008–2009. Source: Data derived from the Brazilian Family Budget Survey—Brazilian Institute of Geography and Statistics (10).

Schooling is also an important socioeconomic indicator to be related to the prevalence of obesity and overweight. In this sense, we looked for an association between educational level and the prevalence of obesity and overweight by sex in adults. Overweight/obese individuals who went up to only elementary school are mostly women, as shown in Figure 9.

## Discussion

Owing to rapid economic growth and urbanization, overweight, obesity, and related diseases have reached drastic levels worldwide. The increasing global prevalence of obesity considers it to be a pandemic. In Brazil, overweight and obesity are also major issues, and there is no single state that is free from population growth in overweight levels. However, this growth is more intense in some areas and more discreet in others.

The percentage of overweight individuals in the Brazilian states was verified, identifying that all of them have high levels of overweight and obesity. The states of Maranhão (44%) and Bahia (45%), are the states with the lowest level of overweight and obesity. The state of Rio Grande do Sul (60%) has the highest percentage of overweight and/or obesity population. The data used in this study are from 2008, but we can verify that the distribution of obesity remained similar over the 10 years (17). Therefore, we could expect that the observations described here are still meaningful.

The world average of adults with a BMI ≥ 25 kg/m^2^ in 2013 was 36.9% for men and 38% for women (18). Thus, we found that all Brazilian states have rates of overweight higher than the world averages, and monitoring the prevalence of overweight and obesity in all populations is extremely important.

We understand that there is an association between geographic regions and nutritional misalignment. Our findings confirm the moderate spatial autocorrelation for Brazilian overweight that show that there is a higher prevalence of obesity and overweight in the Southern, Southeastern, and Midwestern States. There is a similarity between the spatial division found in obesity and overweight levels in relationship to the geopolitical division of Brazil. The regional variation in obesity and overweight levels could be explained by lifestyle factors, dietary patterns, environmental determinants, and socioeconomic status.

The composition of the seven groups reflects the diversity of Brazilian states in terms of food procurement. The states with the lowest rates of overweight and obesity are in Group 3, suggesting that dietary patterns may indeed be related to overweight levels.

The similarity of eating habits among some Brazilian states could be also explained, in part, based on the regional agricultural production (19). The existence of short food supply chains of certain products can influence the eating patterns of a particular locality. We found that food Group 1 concentrates most on the fish acquisition. The states that make up this food group are located in the region of the country that produces about 57% of the national fish output. Groups 2 and 7 comprise the states with the highest milk production in Brazil. The states of Minas Gerais, Rio Grande do Sul, and Paraná (18) have 38% of the dairy purchases.

In addition, visualizing the food acquisition in the state of Rio Grande do Norte (RN) may help explain its different overweight/obesity indices, when compared to the other states in the Northeast region of Brazil. The RN has a close/similar food acquisition to the Brazilian south region. We emphasize that Rio Grande do Norte has greater access to certain foods also available in the south of the country where the population lives mostly in the coastal region.

The identification of seven distinct dietary patterns emphasizes the influence of agribusiness supply chains on regional dietary patterns. Differences in food supply between states occur due to political, economic, logistic, and biophysical factors. It is obviously impossible to make healthy food choices if there is no local pertinent food availability. Because food security is a concept that also involves food availability, identifying the geographical factors that affect food choices is relevant for promoting health and malnutrition eradication (1, 2).

From a public health viewpoint, identifying dietary determinants of weight gain is critical to understanding and reducing the prevalence of obesity. However, although the dietary pattern is not the only determinant of the geographical distribution of overweight/obesity, in some cases, it is extremely related.

Socioeconomic background and food environment can have a strong influence on the nutritional status and health of individuals. Additional evidence can be found in the repeated findings on income and/or education-related obesity growth rates (13, 14, 18). The Brazilian's socioeconomic inequality is certainly a major factor in the development of the patterns we identified in this work (20).

A possible relationship between income and food acquisition profile is visible. With these results, apparently, economic development does not necessarily result in an improvement in the food-related health of Brazilians. In developing countries, where poverty and wealth coexist, obesity should be an indicator of easy access to food, leading us to believe that in the poorest countries, overweight is present in the higher-income classes.

Some studies (21–23) that analyze the relationship between nutritional status and socioeconomic conditions in Brazil, identified differences for men and women. For most countries, female obesity is higher than male obesity. Women are more obese in low-income and middle-income countries, but the gender obesity gap disappears in high-income economies (24). In Brazil, we also found higher levels of overweight and obesity in women.

Many analyses include sex as a clear effect modifier, concluding the association of different directions between education and obesity (25). We found that in Brazil, the overweight/obese individuals who went only up to elementary school are mostly women. Among people with higher levels of education, we find more men with obesity and overweight.

Our evidence supports that sex is a factor that should be considered in developing public policies to overcome obesity and overweight. Among low-income people with overweight/obesity, women are conspicuous. In the highest income quartile, we find more men with obesity and overweight.

We found different relationships between education and nutritional outcomes in Brazilian women, compared to those in men. This finding deserves attention because education not only facilitates access to health information but also helps the cognitive processes associated with the promotion of healthy habits (26). A study in northeastern Brazil found that in males, the risk of overweight is directly associated with a higher educational level, while in females, it is inversely associated (23). Another study (21) confirmed this finding among men, identifying that those with a lower level of education in Brazil were less likely to be obese compared to individuals with a higher school degree.

We understand sex as a potential confounding factor or modifier of the effect of the relationship between socioeconomic and nutritional status. It is important, therefore, to continue the investigation on the effect of sex in the relationship of socioeconomic indicators with the nutritional status of individuals.

## Conclusion

This study provides a set of empirical findings on the relationship between socioeconomic status and the prevalence of obesity and overweight in the geographically spread Brazilian population. Our evidence supports that both age and gender should be considered when designing public policies to fight obesity and overweight.

Overweight and obesity cannot be viewed as an individual problem. It is important to recognize that not everyone has access to a healthy lifestyle and quality food. We understand that socio-geographic factors influence nutritional misalignment in Brazil, and we confirm this hypothesis by identifying a spatial distribution in the country's overweight and obesity population levels. We verified the variations in distributing obesity levels, according to dietary patterns and demographic data.

However, we realize that the geography of obesity and overweight in Brazil is more complex than might have been seen here or previously assumed. The distinct and changing economic, social, and cultural environments that characterize Brazil make the country very diverse, complex, and dynamic in the patterns of the social determinants of obesity. We shall acknowledge that different population groups may have divergent nutritional situations, which depend on multiple variables that transcend those detectable in dietary patterns.

Together, the results of this study indicate that dietary patterns cannot be sentenced as decisive for the high rates of obesity/overweight, as a nutritional evaluation of food standards was not performed. It is possible to consider that the relationships we described between diet and overweight exists; however, a further evaluation of the respective diets is necessary. Moreover, a healthy community is a population that, besides having excellent medical care, also lives in an environment that encourages individuals to carry out a healthy life. An environment that avoids the prevalence of obesity should be based on the availability and supply of food that correspond to a healthy diet and on strong policies that promote general education.

In conclusion, we identified a set of factors that suggest why obesity and overweight may prevail in certain populations. A more global look is needed on this pandemic and how it is distributed in different locations. A limitation of this study was its contingency to updated public data and the last Brazilian bank available to us dated from 2008 to 2009. Comparable and updated information on levels and trends are important for better and continuous use of our results. We hope this research can contribute to developing public policies to adjust the regional production and distribution of food to ensure food in quantity and quality for entire populations.

## Data Availability Statement

Publicly available datasets were analyzed in this study. This data can be found here: https://www.ibge.gov.br/estatisticas/multidominio/condicoes-de-vida-desigualdade-e-pobreza/9050-pesquisa-de-orcamentos-familiares.html?=&amp;t=microdados.

## Author Contributions

CA and GD collected the data and carried out the statistics. KV and HD supported the intellectual and analytical framing of the work. CA and HD wrote the article.

### Conflict of Interest

The authors declare that the research was conducted in the absence of any commercial or financial relationships that could be construed as a potential conflict of interest.
